# Supplementary material for: Species matter for predicting the functioning of evolving microbial communities – An eco-evolutionary model
Source: PLoS One. 2019 Aug 19;14(8):e0218692. doi: 10.1371/journal.pone.0218692 (PMC6699713; doi:10.1371/journal.pone.0218692)
Supplement: S1 Appendix — (PDF) [file pone.0218692.s001.pdf]

S1 APPENDIX

Evolutionary model

The canonical equation of adaptive dynamics is similar but includes a linear effect of population size on evolutionary rates. Both theory and evidence show that evolutionary rates saturate with increasing population sizes and it is assumed here that microbial populations are at high enough densities (for example in the human gut) that evolutionary rates across species should be independent of population size. This approach also assumes that there is no diversification; i.e., that cohesive forces are strong enough to prevent population divergence and only changes in mean phenotype occur.

Steady-state solution for a linear pathway performed by a set of specialists with constant flow

Substrate 1 arrives in the inflow at concentration Q_i, at dilution rate D, and is metabolised by species 1 to generate substrate 2. Substrate 2 is metabolised by species 2 into substrate 3 and so on until the production of the end product, substrate *m*, which is produced by species *m-1* through metabolism of substrate *m-1* and not itself metabolised by any species. Because each substrate is only used by one species, a single index suffices to denote each reaction:

$$\frac{dS_1}{dt} = D(Q_1 - S_1) - J_1 N_1$$

$$\frac{dS_i}{dt} = J_{i-1} N_{i-1} - J_i N_i - DS_i \qquad , for\ i = 2\ to\ m-1$$

$$\frac{dS_m}{dt} = J_{m-1} N_{m-1} - DS_m$$

$$\frac{dN_i}{dt} = c_i J_i N_i - DN_i$$

First, solve steady-state for substrate 1 and species 1, which is unaffected by later steps and is the solution for a single population and single substrate (Hebert et al. 1956).

$$\tilde{S}_1 = \frac{DK_1}{c_1 v_1 - D} \quad , \quad \tilde{N}_1 = c_1(Q_1 - \tilde{S}_1)$$

These solutions apply as long as the dilution rate is below a critical value

$$D < \frac{c_1 v_1 Q_1}{Km_1 + Q_1}$$

above which washout occurs and $N_1 = 0$, $S_1 = Q_1$. We only consider solutions below this threshold. At steady-state, $J_1N_1 = D(Q_1 - S_1)$, therefore the equation for substrate 2 becomes

$$\frac{dS_2}{dt} = D\left(Q_1 - \tilde{S}_1 - \tilde{S}_2\right) - J_2N_2 = 0$$

and by iteration

$$\frac{dS_i}{dt} = D\left(Q_1 - \sum_{j=1\ to\ i} \tilde{S}_j\right) - J_iN_i = 0$$

which yields the solutions for $i > 1$ and $i < m$ of

$$\tilde{S}_i = \frac{DK_i}{c_iv_i - D} \quad , \qquad \tilde{N}_i = c_i(Q_1 - \sum_{j=1\ to\ i} \tilde{S}_j)$$

as long as

$$D < \frac{c_iv_i(Q_1 - Y_i)}{(K_i + Q_1 - Y_i)} \quad , where\ Y_i = \sum_{j=1\ to\ i-1} \tilde{S}_j$$

otherwise $N_i = 0$, $S_i = Q_1 - Y_i$. If the previous reaction does not occur because of washout, then $N_i = 0$ and $S_i = 0$ at steady-state. The steady-state of the final end product that is not used by any bacterial species is either 0 if the pathway terminates before its production or $Q_1 - Y_{m-1}$. Steady-state coexistence is impossible for two species that both specialise on the same substrate, even if they degrade it into separate products, unless all other parameters are identical yielding a neutral equilibrium (Hsu et al. 1977; Smith and Waltman 1995).

The same approach can be used to calculate steady-states for converging and diverging pathways, as long as each substrate is metabolized just by a single specialist population. For converging pathways, the steady-state concentration of the substrate i at the point of convergence depends solely on its rate of conversion to biomass by the species that uses it, as in the linear case. The steady-state abundance of the species using the substrate and subsequent steps is derived by summing the resources arriving from each input pathway:

$$\tilde{N}_i = c_i\left(Q_x - \sum_{j=x\ to\ i} \tilde{S}_j + Q_y - \sum_{j=y\ to\ i} \tilde{S}_j\right)$$

where x is the input substrate for one branch and y is the input substrate for the other, and the standing concentrations of all intermediate substrates are summed. Similarly, diverging pathways can be solved as long as one species uses a single substrate but with enzyme to metabolise it into multiple products.

Alternative measures of functioning

Various metrics of community functioning can be calculated. For example, waste-water treatment, might aim to reduce the concentration of the input substrate $\tilde{S}_1$ in the outflow. $\tilde{S}_1$ declines as the maximum per capita growth rate of bacteria using substrate 1 (c_1v_1) increases, or when K_1 decreases, i.e. when the enzyme has a higher affinity and growth rate saturates at a lower substrate concentration. Introducing a species with higher c_1v_1 or lower K_1 would therefore reduce S_1, which is feasible since this species would be able to outcompete the incumbent species performing this role and invade (Hsu et al. 1977). A simpler way to lower the concentration of S_1 in the outflow is to reduce the dilution rate (fig. 2D). However, if the goal is to decompose the greatest amount of S_1 per unit time, given by $D(Q_1\text{-}\tilde{S}_1)$, rather than low outflow concentration, then an intermediate dilution rate is optimal (fig. S1A), given by:

$$D_{opt} = \frac{c_1v_1(K_1 + Q_1 - \sqrt{K_1(K_1 + Q_1)}\,)}{K_1 + Q_1}$$

In a bioreactor or animal gut, the aim might be to increase the production of an intermediate substrate. For example, the main benefits of fermentation of fibre in the human gut derive from short-chain fatty acids, which themselves can be fermented to other products. The steady-state concentration $\tilde{S}_i$ is independent both of the concentration of input resource, Q_1 and of earlier steps, as long bacteria that perform those steps persist. Instead $\tilde{S}_i$ increases as c_iv_i declines or K_i increases, i.e. when the species degrading substrate i grows more slowly. This is harder to target by replacing existing species since it requires that the species degrading the focal substrate is replaced by a slower-growing species, but such a species could not invade (unless it had compensating advantages not included in this model). A more practical biological intervention is to introduce mortality to species i. This is feasible by reducing the abundance of species converting the substrate to other products. For example, a per capita mortality rate δ would increase substrate i concentration to:

$$\tilde{S}_i = \frac{(D + \delta)K_i}{c_iv_i - (D + \delta)}$$

Alternatively, the dilution rate can be tuned to be slow enough to allow bacteria performing all preceding reactions to persist, but fast enough to prevent species i from persisting (fig. 2), i.e. at the threshold defined by $D < \frac{c_i v_i (Q_1 - Y_i)}{(K_i + Q_1 - Y_i)}$. If the goal is to maximize the amount harvested per unit time rather than outflow concentration, a higher dilution rate can be optimal if $max(D\tilde{S}_i)$ for D below the threshold is less than $max(D(Q_1 - Y_i))$ for higher D (Figure S1B). Varying dilution rate, i.e. transit times, is mechanism used by animal hosts of gut microbiota, via hormonal feedbacks triggered by levels of bacterial metabolites in the gut.

In applications such as biogas generation, the aim might be to increase production of the end product. The outflow concentration of the final substrate increases with the concentration of input resource (fig. 2), or by reducing K_i or increasing $c_i v_i$ of species performing any intermediate reaction. Given a choice, the impact of reducing the K_i of just one reaction by an absolute amount would be greatest for the reaction with the highest value of $D/(c_i v_i - D)$, and the impact of increasing per capita growth rate of the bacterium would be greatest for the reaction with the highest value of $DK_i/(c_i v_i - D)^2$ (i.e. the change in $\tilde{S}_m$ for a small change in $c_i v_i$). Another approach would be to replace two species feeding on adjacent substrates in the pathway with a species that skips the intermediate step in the pathway (i.e. converts S_i to S_{i+2} directly). This would enhance production of the final substrate as long as $DK/(cv-D)$ for reaction S_i to S_{i+2} is less than $DK/(cv-D)$ for reaction S_i to S_{i+1} so that the new species can invade. This condition also ensures that the new $\tilde{S}_i$ is less than the sum of $\tilde{S}_i$ and $\tilde{S}_{i+1}$ previously, which increases $\tilde{S}_m$ by reducing Y_m (the total concentration of all earlier substrates). The production rate of the final substrate, $D(Q_1 - Y_m)$, is greatest at a dilution rate intermediate between 0 and the threshold $D < \frac{c_i v_i (Q_1 - Y_i)}{(K_i + Q_1 - Y_i)}$ when $i=m-1$ (Fig. S1B). Other measures of metabolic functioning such as total production of a subset of metabolites or a target cocktail of relative concentrations could be derived from the same expression. As well as metabolic functioning, the equations can be used to derive ecological measures of functioning such as total biomass, productivity of biomass production per unit time, respiration rates and the abundances of particular key species.

The above predictions would change if assumptions changed. For example, with product inhibition, the rate of each reaction will depend on reactions further down the chain as well. This could be added to the model as required. Also, parameter values are assumed to be independent: optima would differ if there are trade-offs, for example between v and c (Gudelj et al. 2007).

Single generalist species with 2 input substrates

The Monod model for a single generalist growing on two input resources is:

$$\frac{dS_i}{dt} = D(Q_i - S_i) - J_i N_1$$

$$\frac{dN}{dt} = (c_1 J_1 + c_2 J_2)N - DN$$

This is not analytically solvable assuming a Monod function for growth. A version of the model with a linear growth rate instead of saturating with substrate concentration, i.e. replacing $J_i = kcat_i E_i S_i$ is solvable, although the general solution is too complex to be useful (solutions found using SageMathCloud). The most complex version that yields a simple analytical result assumes all parameters are equal for both substrates except the input concentrations (Q_1 and Q_2) and the conversion parameters (c_1 and c_2). Then:

$$\tilde{S}_i = \frac{2DQ_i}{v(c_1 Q_1 + c_2 Q_2)} \quad , \quad \widetilde{N}_1 = \frac{(c_1 v Q_1 + c_2 v Q_2 - 2D)}{v}$$

Analytical results were used to cross-check the simulation results presented in figure 4.

Single generalist species with 1 input and 1 derived substrate

The model of a single generalist metabolizing 1 input substrate into a derived product that can also be metabolised is:

$$\frac{dS_1}{dt} = D(Q_1 - S_1) - J_1 N_1$$

$$\frac{dS_2}{dt} = D(-S_i) - J_2 N_2$$

$$\frac{dN}{dt} = (c_1 J_1 + c_2 J_2)N - DN$$

Again, the Monod model cannot be solved, but useful expressions can be obtained by assuming a linear growth function. In the simplest case of a model assuming all parameters are equal and $E_1 = E_2 = 0.5$, the steady-state concentrations of the input substrate ($\tilde{S}_1$) and the derived substrate ($\tilde{S}_2$) are:

$$\tilde{S}_{1,gen} = \frac{cvQ - \sqrt{c^2v^2Q^2 - 2cvQD}}{cv} \quad , \quad \tilde{S}_{2,gen} = \frac{2D - cvQ + \sqrt{c^2v^2Q^2 - 2cvQD}}{cv}$$

with positive real solutions when $cvQ > 2D$ and $cvQ < 2D + \sqrt{c^2v^2Q^2 - 2cvQD}$. Compared to the equivalent steady-state for 2 specialists ($\tilde{S}_{1,spec} = \tilde{S}_{2,spec} = D/cv$), the sum $\tilde{S}_1 + \tilde{S}_2$ is the same in both cases *=2D/cv*. However, generalist solution for the steady-state concentration of the input substrate is always higher than the specialist solution ($\tilde{S}_{1,gen} > \tilde{S}_{1,spec}$) whereas for the steady-state concentration of the derived substrate is always less than the specialist solution ($\tilde{S}_{2,gen} < \tilde{S}_{2,spec}$). This is because the generalist devotes half the enzyme to the two substrates. Therefore reaction 1 is performed less effectively than by a specialist (hence higher steady-state concentration of substrate 1). The supply rate of substrate 2 is consequently lower, yet metabolism of substrate 2 occurs at a relatively high rate because enzyme 2 is sustained by growth on substrate 1. Even in the simplest model with all other things being equal, therefore, a generalist feeding on two resources in a linear pathway functions differently from 2 specialists with the same enzymes: the solution depends on how enzymes are packaged together within cells.

Coexistence criterion for 2 generalists on 2 substrates for a pathway with one input and one derived substrate. The threshold quantity for coexistence (equivalent to equation 6 for the parallel pathway) is:

$$E_{thresh} = \frac{c_1(c_1v_1v_2Q_1 - v_2D)}{c_1(c_1 + c_2)v_1v_2Q_1 - c_1v_1D - (c_1 + c_2)v_2D}$$

Evolution of single species on 1 input and 1 derived substrate.

$$\tilde{S}_i = \frac{D}{c_iv_i}$$

$$\tilde{E}_1 = \frac{(c_1v_1Q_1 - D)}{(c_1v_1Q_1 - D + c_2v_1Q_1 - D(c_1v_1 + c_2v_2)/c_1v_2)}$$

$$\tilde{N} = \frac{(c_1v_1Q_1 - D + c_2v_1Q_1 - D(c_1v_1 + c_2v_2)/c_1v_2)}{v_1}$$

Growth is sustained as long as growth on the input substrate is viable (i.e. $\widetilde{NE}_1$> 0 as long as $Q_1 > D/c_1v_1$). Enzyme is then allocated to the derived substrate as long as $Q_1 >$

$D(c_1 v_1 + c_2 v_2)/c_1 c_2 v_1 v_2$, which it must be since $Q_1 > D/c_1 v_1$ and $(c_1 v_1 + c_2 v_2)/c_2 v_2$ has to be greater than 1. There is therefore no steady-state solution for growth on substrate 1 where enzyme is not allocated to the derived substrate 2 as well. $\tilde{E}_1$can take values above or below 0.5, however, depending on the inequality implied by E_{thresh}. Further insight is obtained from a simpler model assuming that all enzymatic and conversion parameters are the same for substrate 1 and 2. In this case, $\tilde{E}_1 = (cvQ - D)/(2cvQ - 3D)$, which is always >0.5, showing that there is an inherent benefit towards specializing on the input resource, even if the growth yield on both substrates is equivalent.

Evolution in 2 species on 2 substrates. The neutral stability of a 2-specialist solution can be demonstrated by considering invasion of a new genotype with starting density N and enzyme allocation E_1. Its per capita growth rate is given by

$$\frac{dN}{Ndt} = c_1 v_1 E_1 S_1 + c_2 v_2 (1 - E_1) S_2 - D$$

Substituting in the steady-state solution for 2 specialists, $S_1 = D/c_1 k_1$ and $S_2 = D/c_2 k_2$, yields:

$$\frac{dN}{dt} = c_1 v_1 E_1 D/c_1 v_1 + c_2 v_2 (1 - E_1) D/c_2 v_2 - D$$

$$= E_1 D + (1 - E_1) D - D$$

$$= 0$$

Any set of enzyme allocations that yield a positive 2-species solution, which always result in the same steady-state substrate concentrations, has neutral stability against invasion: i.e. with stochasticity an optimal generalist could drift into the population.

Coexistence of 3 species on 3 substrates

Although the full ecological model with linear growth function could not be solved analytically for 3 generalist species, it was explored analytically via two approaches. First, a simpler model assuming that each species devotes half its enzyme to 2 substrates, in a complementary way so each substrate is used by 2 species, could be solved and confirmed the

simulation results. The only solution with 3 species surviving had steady-state concentrations equal to those with a specialist community. For example, with a parallel pathway

$$N_1 = \frac{cv(Q_1 + Q_2 - Q_3) - D}{v}$$

$$N_2 = \frac{cv(Q_1 - Q_2 + Q_3) - D}{v}$$

$$N_3 = \frac{cv(Q_2 - Q_1 + Q_3) - D}{v}$$

$$S_1 = S_2 = S_3 = \frac{D}{cv}$$

whereas steady-state substrate concentrations differed from the specialist case when fewer species survive. For example, when species 1 alone survives then

$$S_1 = \frac{2DQ_1}{cv(Q_1 + Q_2)}$$

$$S_2 = \frac{2DQ_2}{cv(Q_1 + Q_2)}$$

$$S_3 = Q_3$$

and a long expression with square roots of polynomial expressions defines substrate concentrations when 2 species survive (not shown).

Second, by analogy to the 2-species case, the optimum enzyme allocation for an evolving single generalist species for each 3-substrate pathway could be calculated and used to interrogate conditions for coexistence. Similarly to the 2-species case, all 3 species coexisted when one and just one species has $E_1 > E.thresh_1$, a second species has $E_2 > E.thresh_2$ and a third has $E_3 > E.thresh_3$. However, additional simulations display 3-species coexistence under the less restrictive condition that each of the 3 species had enzyme allocations above a different threshold (but potentially one species has allocation above 2 of the thresholds). Whether this condition yielded coexistence or not depended on how close to the optimal allocation each species was and how similar to each other they were. When this criterion was not met, i.e. there was no species with an enzyme allocation above the optimum for one or two of the substrates, then fewer than 3 species always survived (or 3 species were present after 20000 generations but the solution had not yet converged on the steady-state).

Gut model

The non-evolving generalist community provided more robust functioning than the specialist community. Specialist pathways were more like to break at intermediate steps (66/1000 cases) than generalist pathways (0/1000 cases). Substrates at converging point in the pathways, acetate, lactate, and butyrate were produced more consistently in both community types because multiple independent inputs must break to prevent their formation: they were only absent in 6/1000 to 7/1000 trials. The chance of failing to produce propionate or waste gas was higher in specialist communities (29 and 30/1000 cases respectively) because they derive from a single intermediate. Generalist communities only failed to produce all 3 terminal substrates when the whole community failed.

The focus for studies of gut functioning are the short-chain fatty acids (SCFA) acetate, butyrate and propionate. Production of these metabolites feeds into appetite regulation, immune functioning and disease protection in the gut, and each SCFA has a different role supplying energy to particular organs of the body. The total SCFA concentration did not differ consistently between specialist and generalist matched runs: acetate, as an intermediate metabolite, tends to decrease in generalists whereas terminal metabolites butyrate and propionate increase (fig. 5). However, there was on average 29% discrepancy between total SCFA concentration between specialists and generalist, meaning that packaging of enzyme did affect this quantity, but in opposite directions depending on the value of other parameters. Generalist communities have significantly lower SCFA when fewer species survive and when more gas is produced: sets of enzyme parameters that reduce production of gas (e.g. more lactate production, more butyrate conversion from acetate) increase the total SCFA.

Some aspects of functioning were relatively fixed, however, irrespective of enzyme parameters and packaging. Each SCFA has a different physiological function in the body, so I also compared the ratio of each SCFA relative to total SCFA. In specialist communities, these are predictable and conform to simple relationships: the ratio of propionate to butyrate is approximately 1:3 if all pathways persist, 1:1 if only production from lactate persists and 0:1 if the lactate pathway is absent but acetate is produced. More generally, these simple relationships are found whenever the profitability of the multiple pathways from a single intermediate substrate are the same – irrespective of whether performed by specialists or generalists. Specific outcomes depend both on the packaging of different enzymes and on enzyme kinetic parameters, which in these runs varied across metabolic steps.

In the specialist communities, the production rates of acetate and lactate via the input pathways are the same, because of the symmetry of the pathways and the assumption that 50% of glucose metabolized is converted to lactate and 50% to acetate. If the production rate of acetate and lactate in turn is x, then the amounts of lactate converted to propionate, butyrate and acetate will be $x/3$ each. So now the production rate of acetate is $4x/3$. Therefore, butyrate will be produced from acetate proportional to $2x/3$, as will gas. The total production rate of butyrate is therefore proportional to $x/3 + 2x/3 = x$. Consequently, if all steps in the pathway can persist, the ratios of butyrate to propionate will be 3:1, but 1:1 if only the lactate pathways persist and 0:1 if only the acetate pathway persists.

Additional references:

Hsu SB, S Hubbell & P Waltman 1977 A Mathematical Theory for Single-Nutrient Competition in Continuous Cultures of Micro-Organisms *SIAM J. Appl. Math.* 32:366
