# Supplementary figures and images for: Species matter for predicting the functioning of evolving microbial communities – An eco-evolutionary model
Source: PLoS One. 2019 Aug 19;14(8):e0218692. doi: 10.1371/journal.pone.0218692 (PMC6699713; doi:10.1371/journal.pone.0218692)

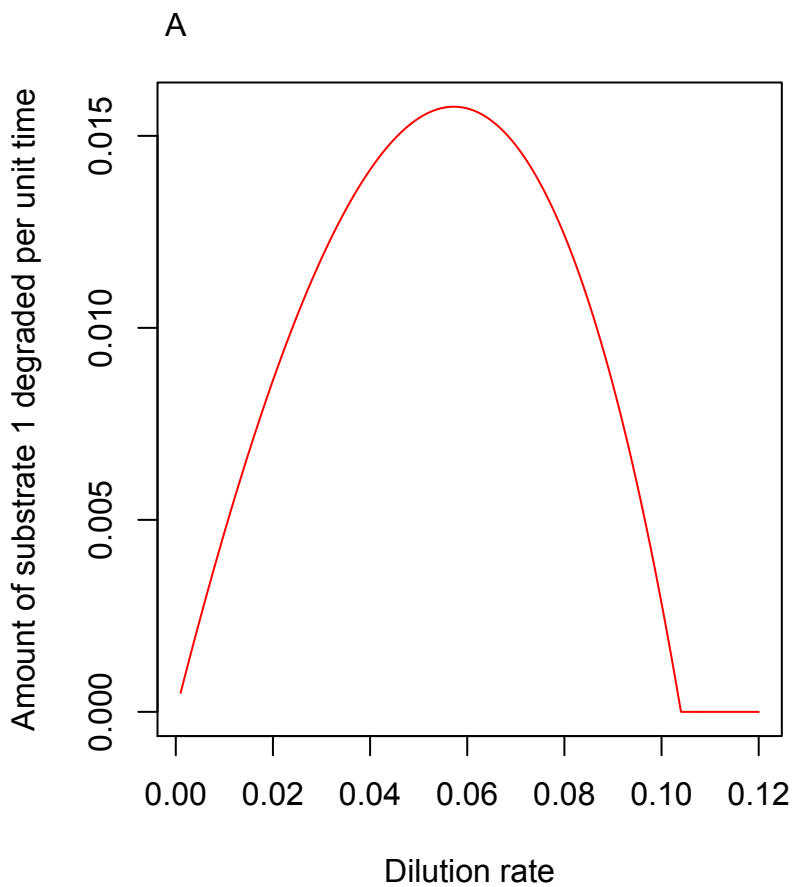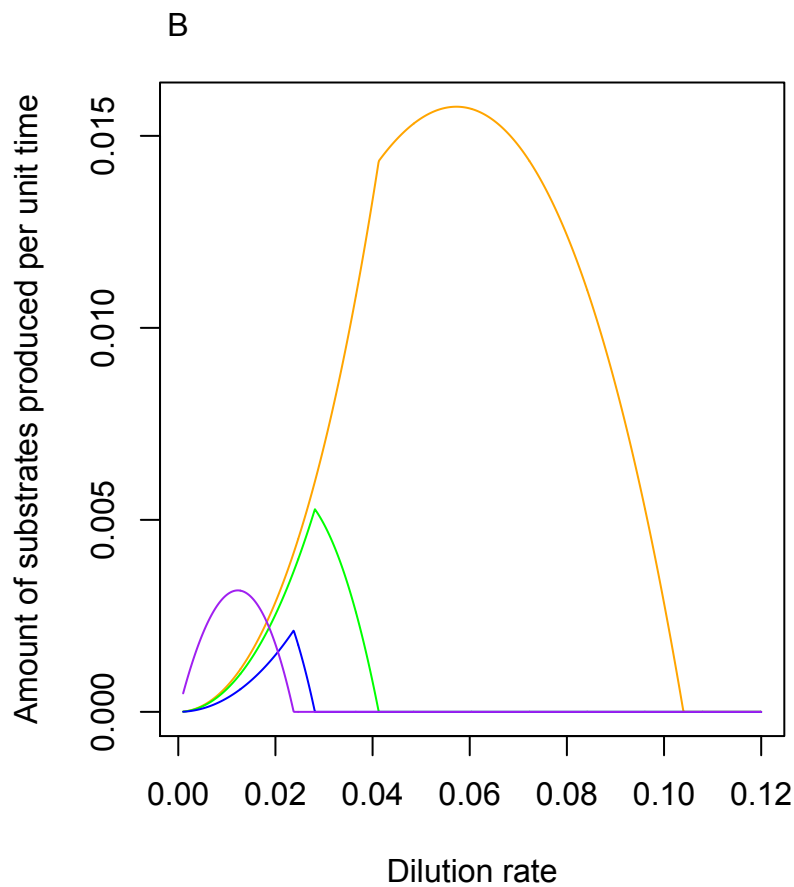

Supplement: S1 Fig — For a linear pathway of 4 specialists degrading a single input resource: (A) the amount of substrate 1 degraded per unit time, i.e. D(Q1−S˜1); (B) The amount of each derived substrate produced per unit time, which is DS˜i below the threshold for washout of species i and D(Q1- Yi) above the threshold. Parameter values were: input substrate concentration, Q1 = 0.5 (in D and E); all v = 0.2; all K = 1; values of c chosen from a normal distribution with mean 1 and sd 0.2, = 1.56, 0.80, 0.89, 1.45 for each reaction in turn. Note the maximum production rate of substrate 2 (orange) occurs at a higher dilution rate than the threshold for persistence of species 2 (which is at the inflection point on the orange curve), but for all other substrates the optimum dilution rate is at the threshold for persistence of the corresponding species. (PDF) [file pone.0218692.s002.pdf]

**A)**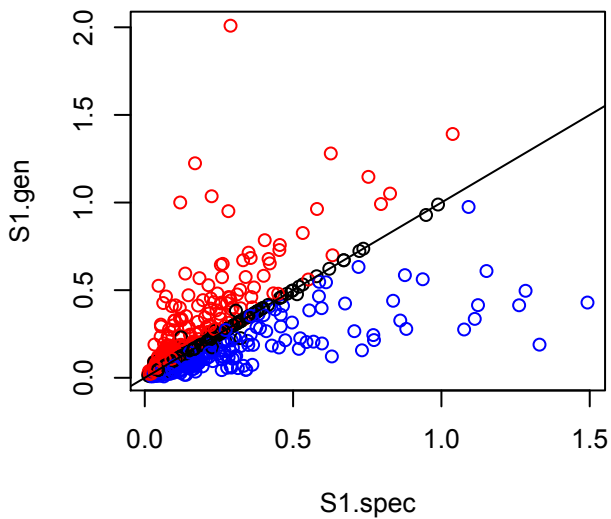**B)**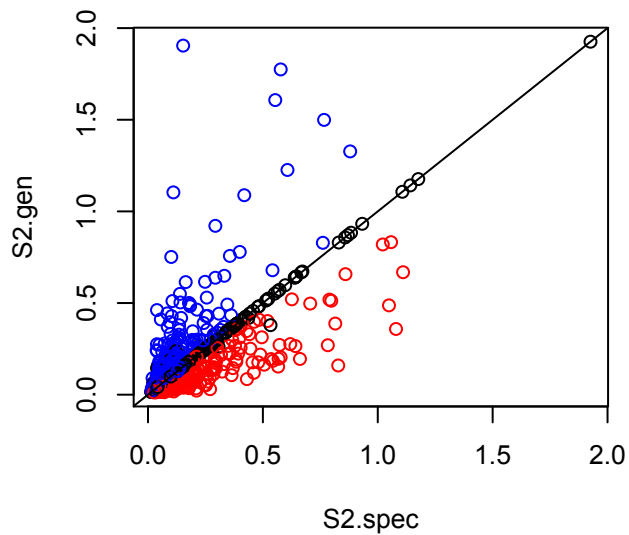**C)**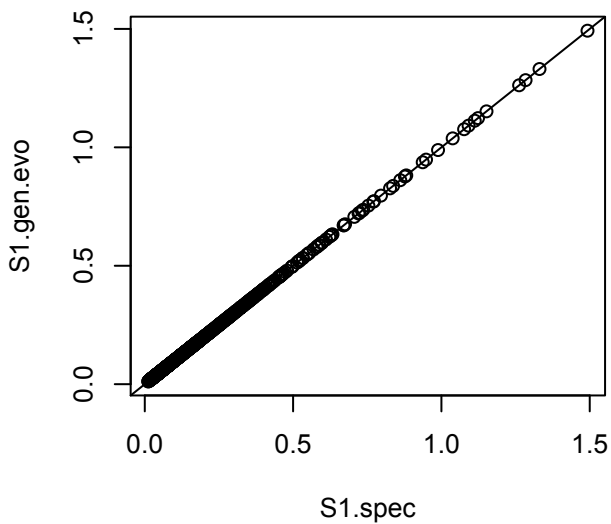**D)**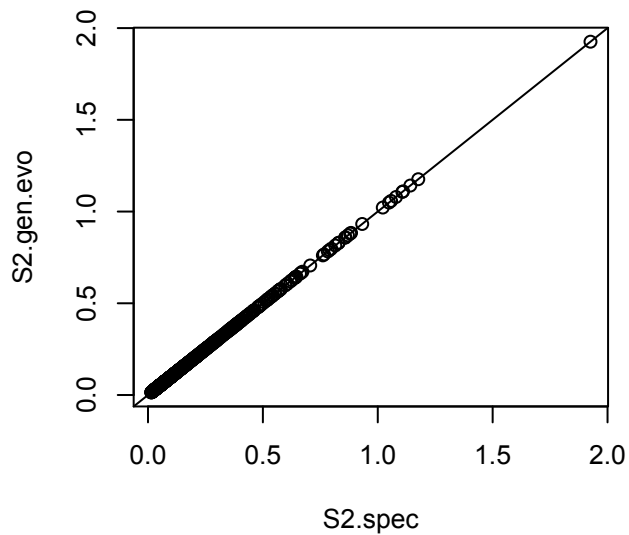

Supplement: S3 Fig — Steady-state concentrations of substrate 1 (A) and 2 (B) for 2 specialists (x-axis) versus 2 generalists (y-axis). Red = just species 1 survives; blue = just species 2 survives; black = both species survive. Species 1 is defined as the species allocating most enzyme to substrate 1, i.e. E11>E12. Steady-state concentrations for substrates 1 (C) and 2 (D) when the generalist species are able to evolve during the simulation. Each point represents a separate run with parameter values chosen from uniform distributions (c = 0.13 to 1, v = 0.13 to 1, D = 0.01 to 0.05, E = random partition of 1.0). Simulations ran for 20000 time units. (PDF) [file pone.0218692.s004.pdf]

**A)**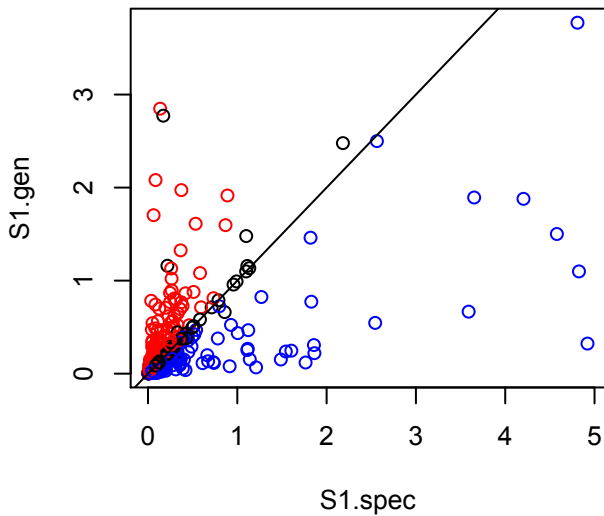**B)**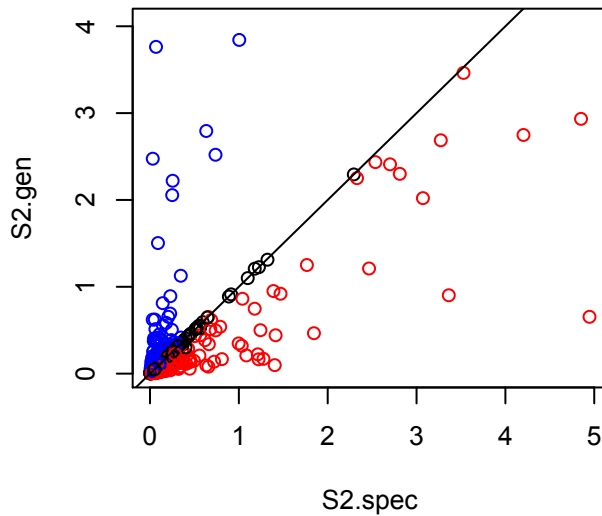**C)**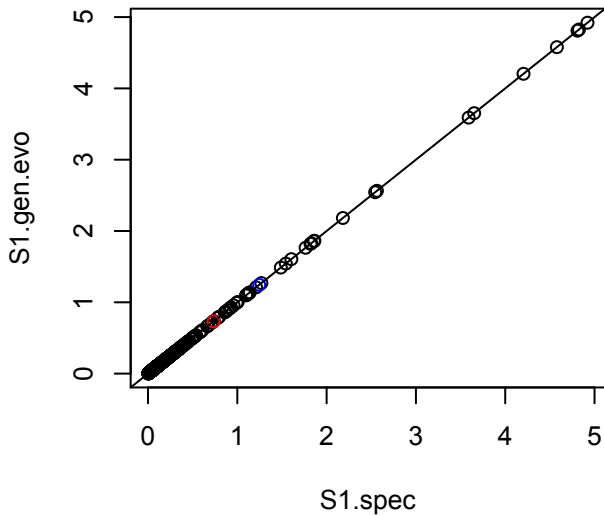**D)**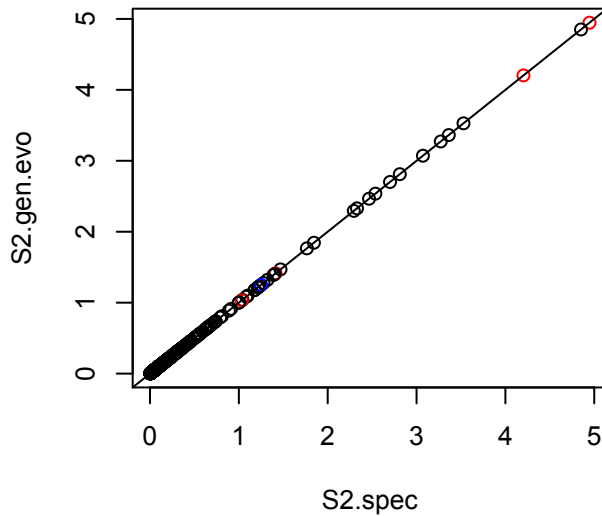

Supplement: S4 Fig — Steady-state concentrations of substrate 1 (A) and 2 (B) for 2 specialists (x-axis) versus 2 generalists (y-axis). Red = just species 1 survives; blue = just species 2 survives; black = both species survive. Species 1 is defined as the species allocating most enzyme to substrate 1, i.e. E11>E12. Steady-state concentrations for substrates 1 (C) and 2 (D) when the generalist species are able to evolve during the simulation. Each point represents a separate run with parameter values chosen from uniform distributions (c = 0.13 to 1, v = 0.13 to 1, D = 0.01 to 0.05, E = random partition of 1.0). Simulations ran for 20000 time units. (PDF) [file pone.0218692.s005.pdf]

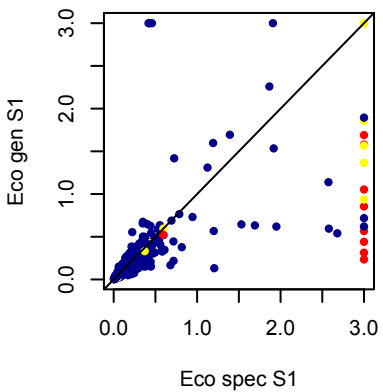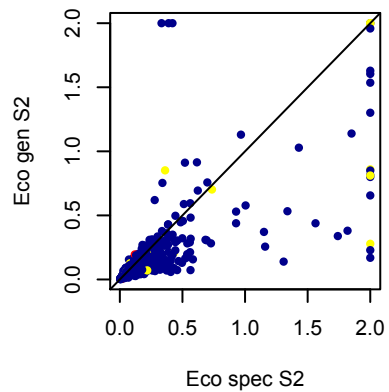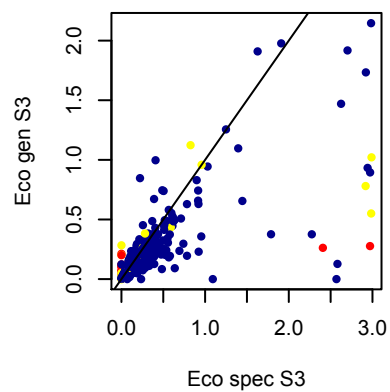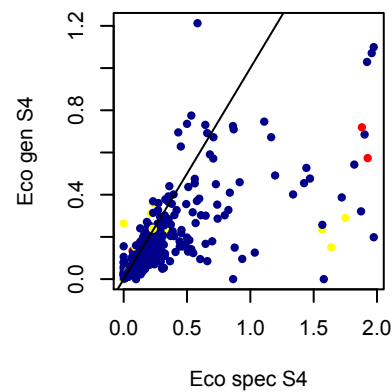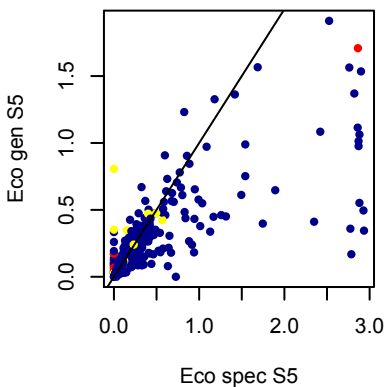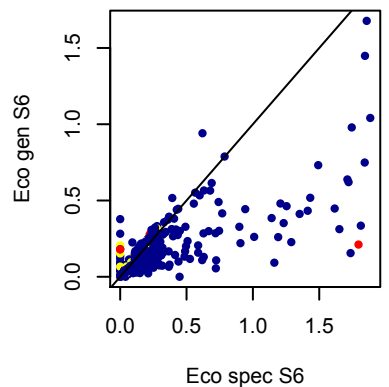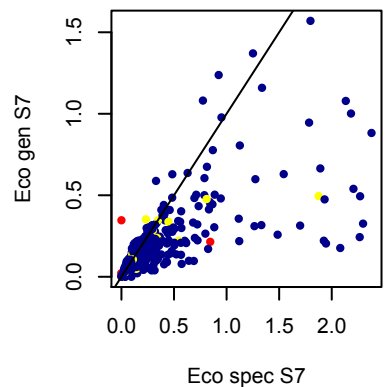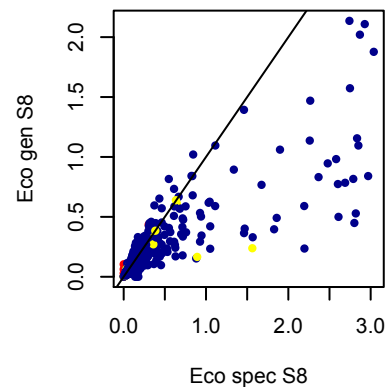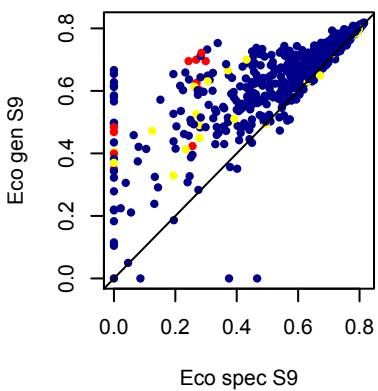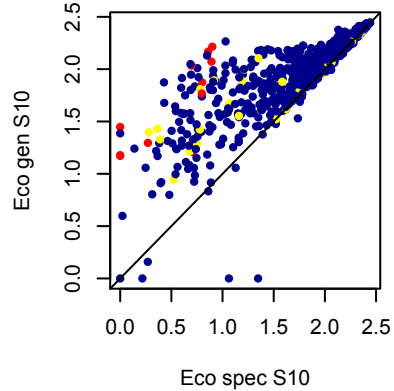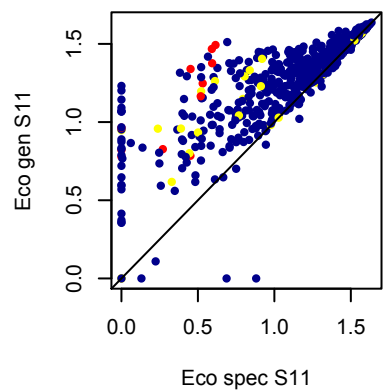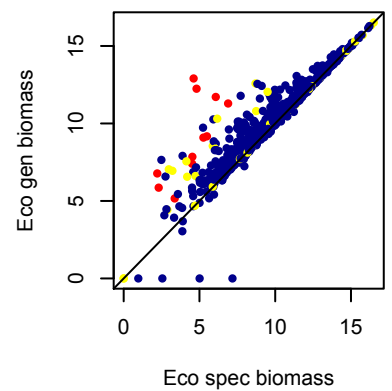

Supplement: S5 Fig — Steady-state concentrations of substrates and biomass for specialists (x-axis) versus non-evolving generalists (y-axis). Blue = more species survive in specialist run; yellow = same number of species survive; red = more species survive in generalist run. Each point represents a separate run with parameter values chosen from uniform distributions (c = 0.13 to 1, v = 0.13 to 1, d = 0.01 to 0.05, E = random partition of 1.0). Simulations ran for 20000 time units. (PDF) [file pone.0218692.s006.pdf]

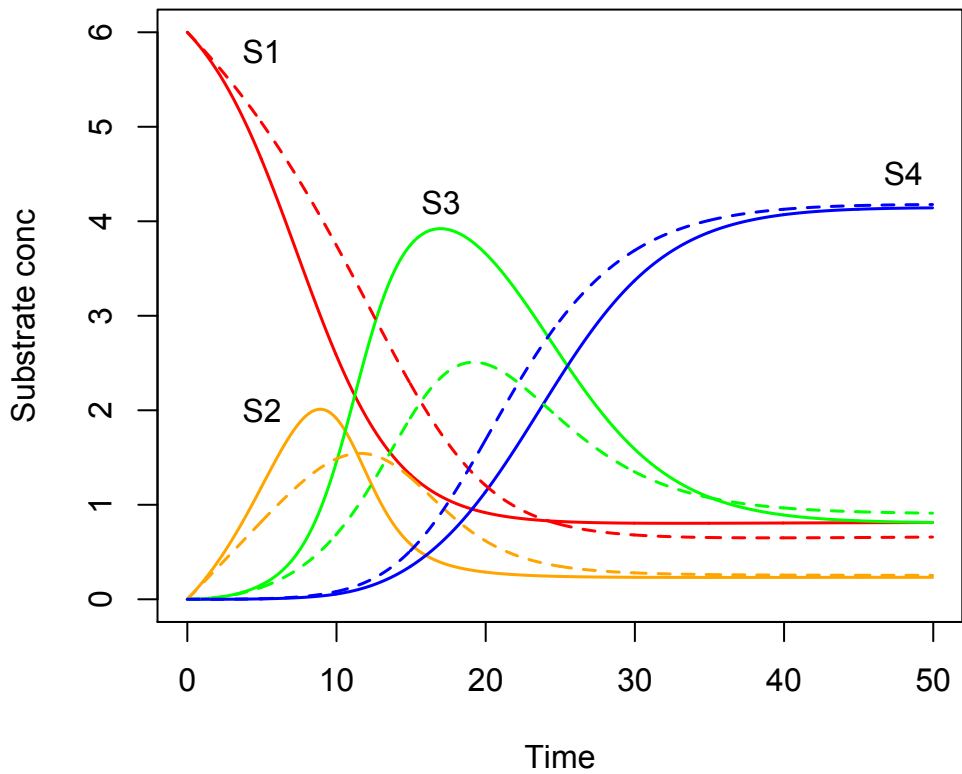

Supplement: S6 Fig — Similar outcomes were observed for the 8-species community but a 3-species systems is shown here for clearer visualization. Parameter values for this example: c1 = 0.20, c2 = 0.33, c3 = 0.23, v1 = 0.28, v2 = 0.57, v3 = 0.24, D = 0.045, Q1 = 6, species 1 E11 = 0.04, E21 = 0.36, E31 = 0.60, species 2 E12 = 0.65, E22 = 0.01, E32 = 0.34, species 3 E13 = 0.32, E23 = 0.63, E33 = 0.05. (PDF) [file pone.0218692.s007.pdf]

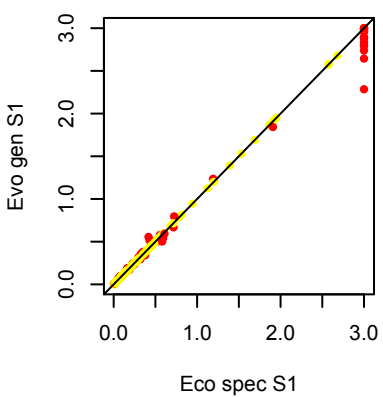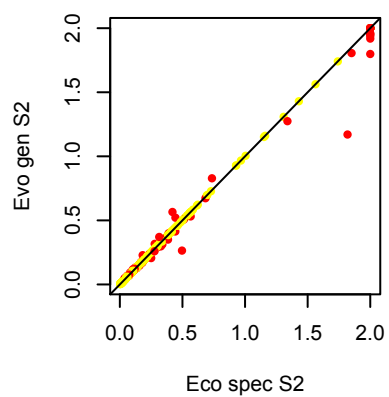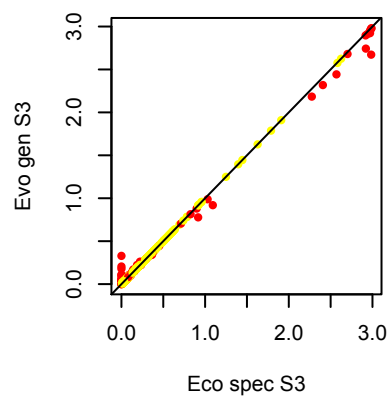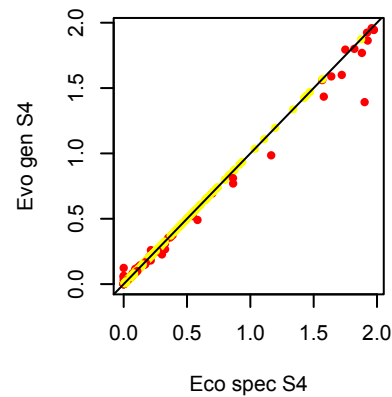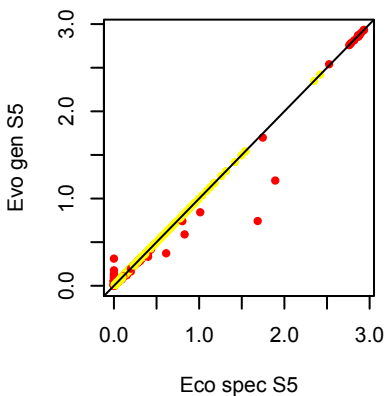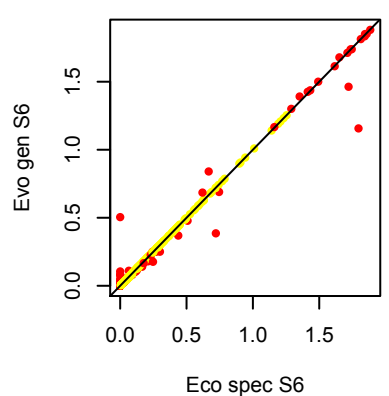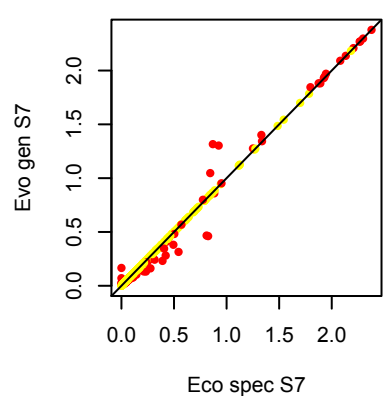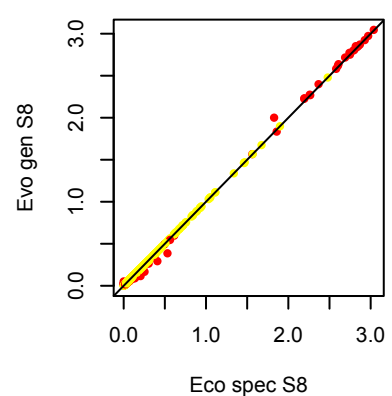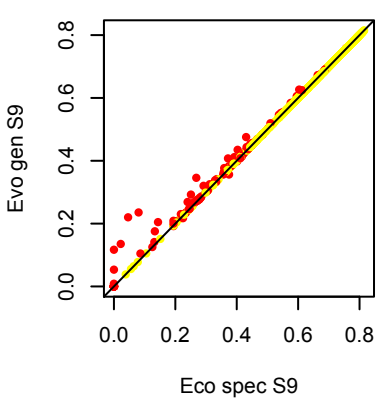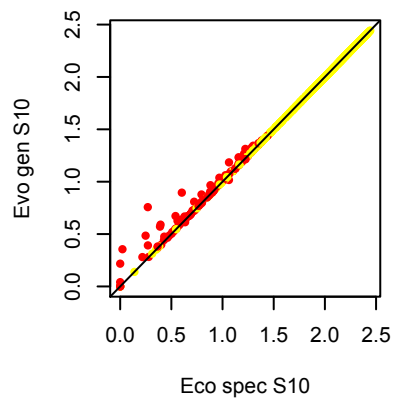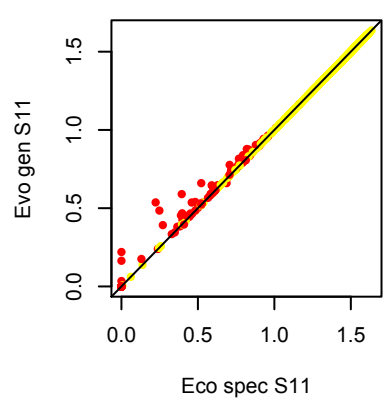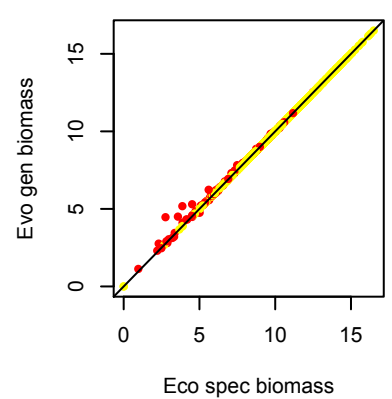

Supplement: S7 Fig — Steady-state concentrations of substrates and biomass for specialists (x-axis) versus evolving generalists (y-axis). Blue = more species survive in specialist run; yellow = same number of species survive; red = more species survive in generalist run. Each point represents a separate run with parameter values chosen from uniform distributions (c = 0.13 to 1, v = 0.13 to 1, D = 0.01 to 0.05, E = random partition of 1.0). Simulations ran for 20000 time units. (PDF) [file pone.0218692.s008.pdf]

Euclidean distance finish

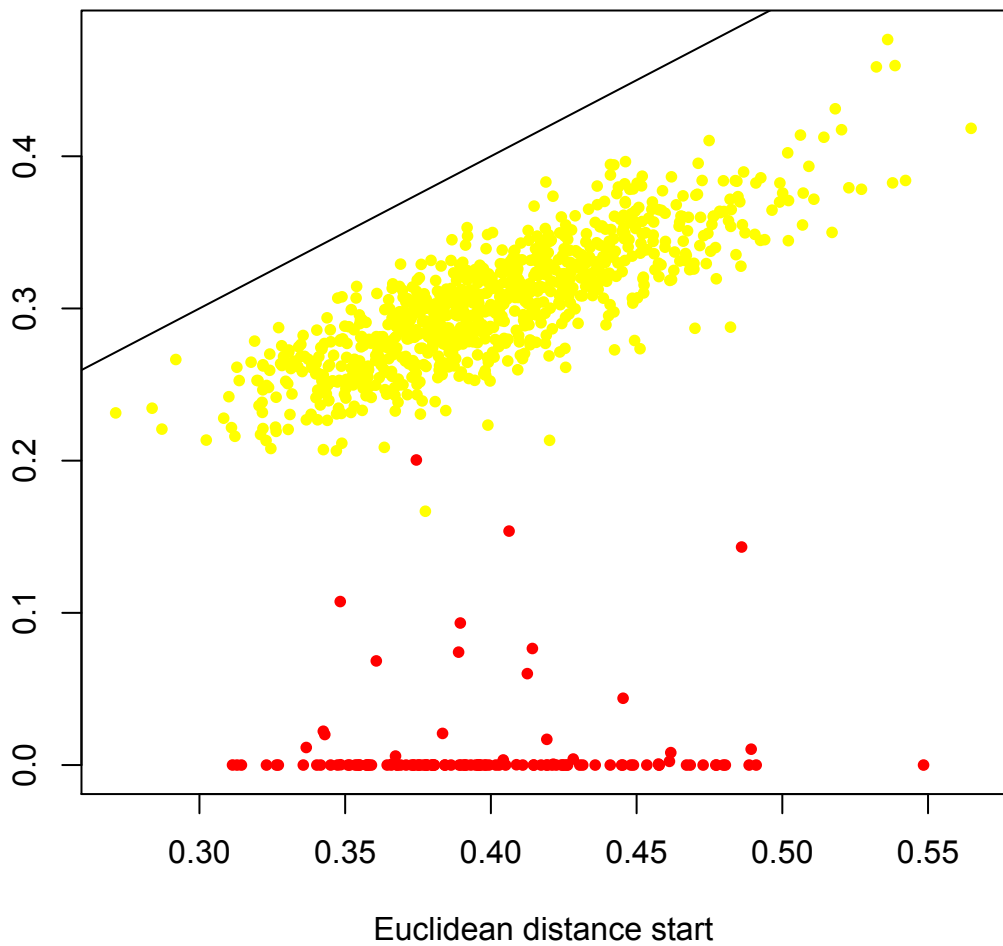

Supplement: S8 Fig — Red = not all specialists survived in the specialist run. Yellow = same number of species survived in the specialist and evolving generalist runs. The 1:1 line is shown: in every run, the species are more similar in their enzyme profile by the end than at the start. (PDF) [file pone.0218692.s009.pdf]

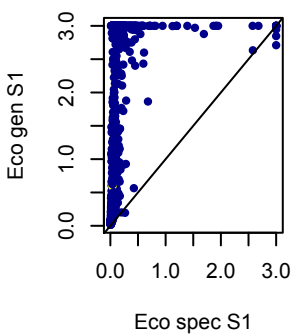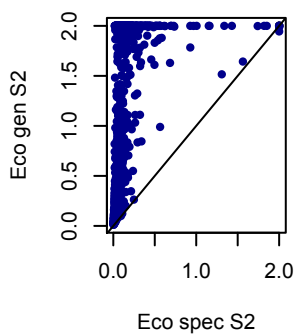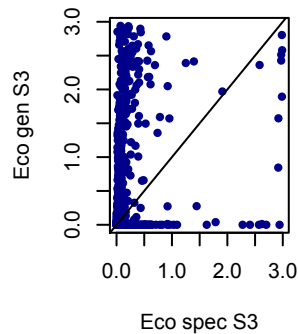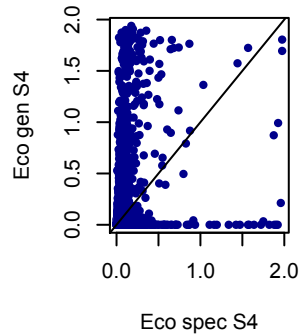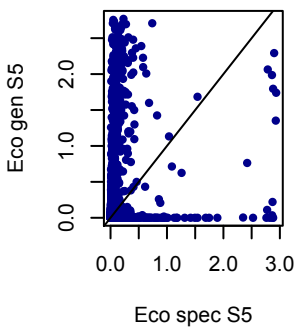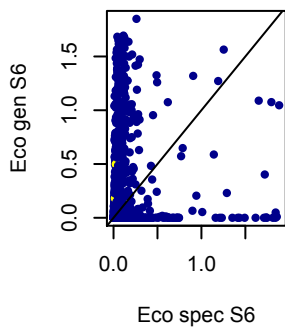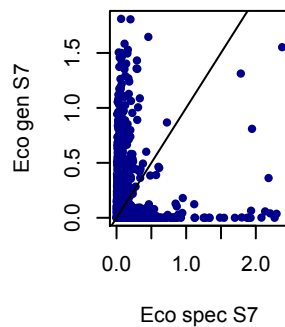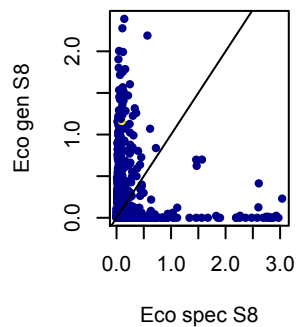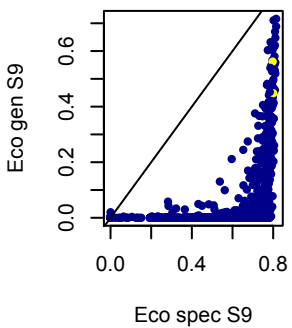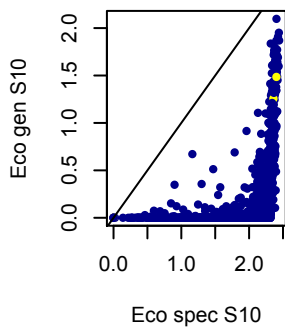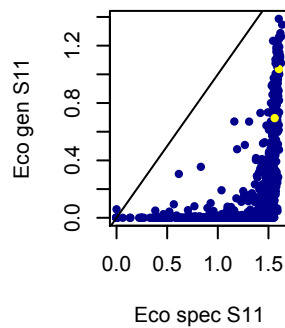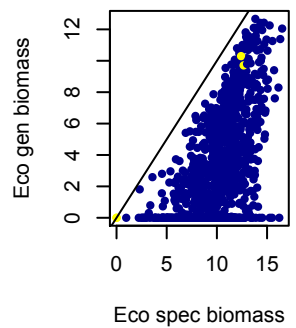

Supplement: S9 Fig — Steady-state concentrations of substrates and biomass for specialists (x-axis) versus non-evolving generalists (y-axis). Blue = more species survive in specialist run; yellow = same number of species survive; red = more species survive in generalist run. Each point represents a separate run with parameter values chosen from uniform distributions (c = 0.13 to 1, v = 0.13 to 1, D = 0.01 to 0.05, E = random partition of 1.0). Simulations ran for 20000 time units. (PDF) [file pone.0218692.s010.pdf]

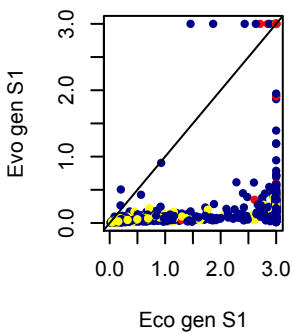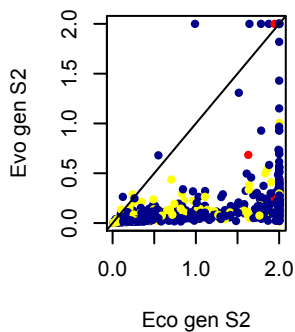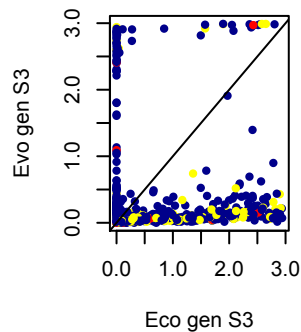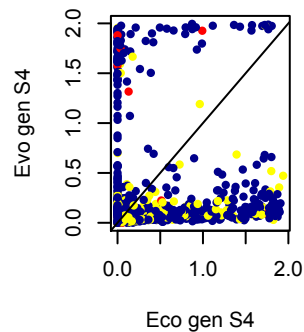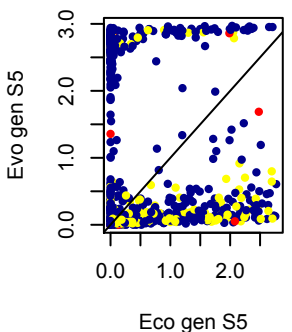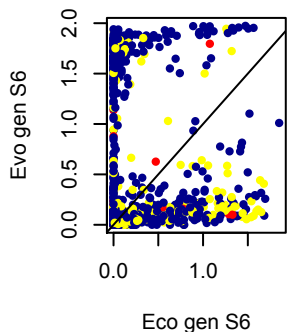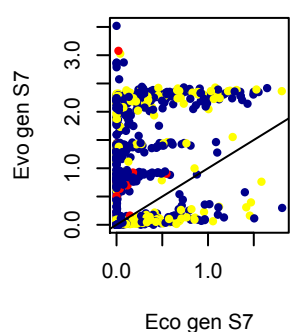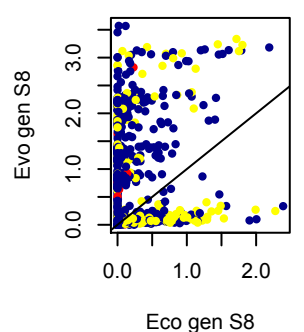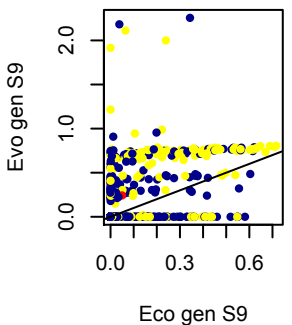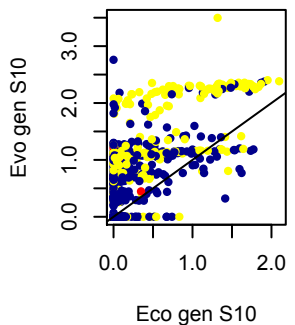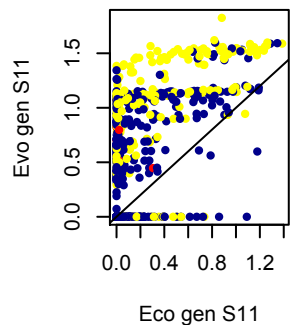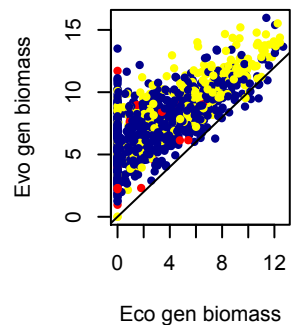

Supplement: S10 Fig — Steady-state concentrations of substrates and biomass for specialists (x-axis) versus evolving generalists (y-axis). Blue = more species survive in specialist run; yellow = same number of species survive; red = more species survive in generalist run. Each point represents a separate run with parameter values chosen from uniform distributions (c = 0.13 to 1, v = 0.13 to 1, D = 0.01 to 0.05, E = random partition of 1.0). Simulations ran for 20000 time units. (PDF) [file pone.0218692.s011.pdf]
