# Supplementary material for: Species matter for predicting the functioning of evolving microbial communities – An eco-evolutionary model
Source: PLoS One. 2019 Aug 19;14(8):e0218692. doi: 10.1371/journal.pone.0218692 (PMC6699713; doi:10.1371/journal.pone.0218692)
Supplement: S2 Fig — Each point represents a separate run with parameter values chosen from uniform distributions (c = 0.1 to 1, v = 0.1 to 1, K = 0.01 to 5, D = 0.01 to 2, E = random partition of 1.0). Input concentration of substrate 1 was held fixed at 5 units. Parameters were fixed to be the same for substrate 1 and 2 except for the parameter varied in each panel as described in titles. The same parameters were used for matching specialist and generalist runs represented by a single point in order to compare the effects of enzyme packing into species. Simulations ran for 2000 time units. (PDF) [file pone.0218692.s003.pdf]

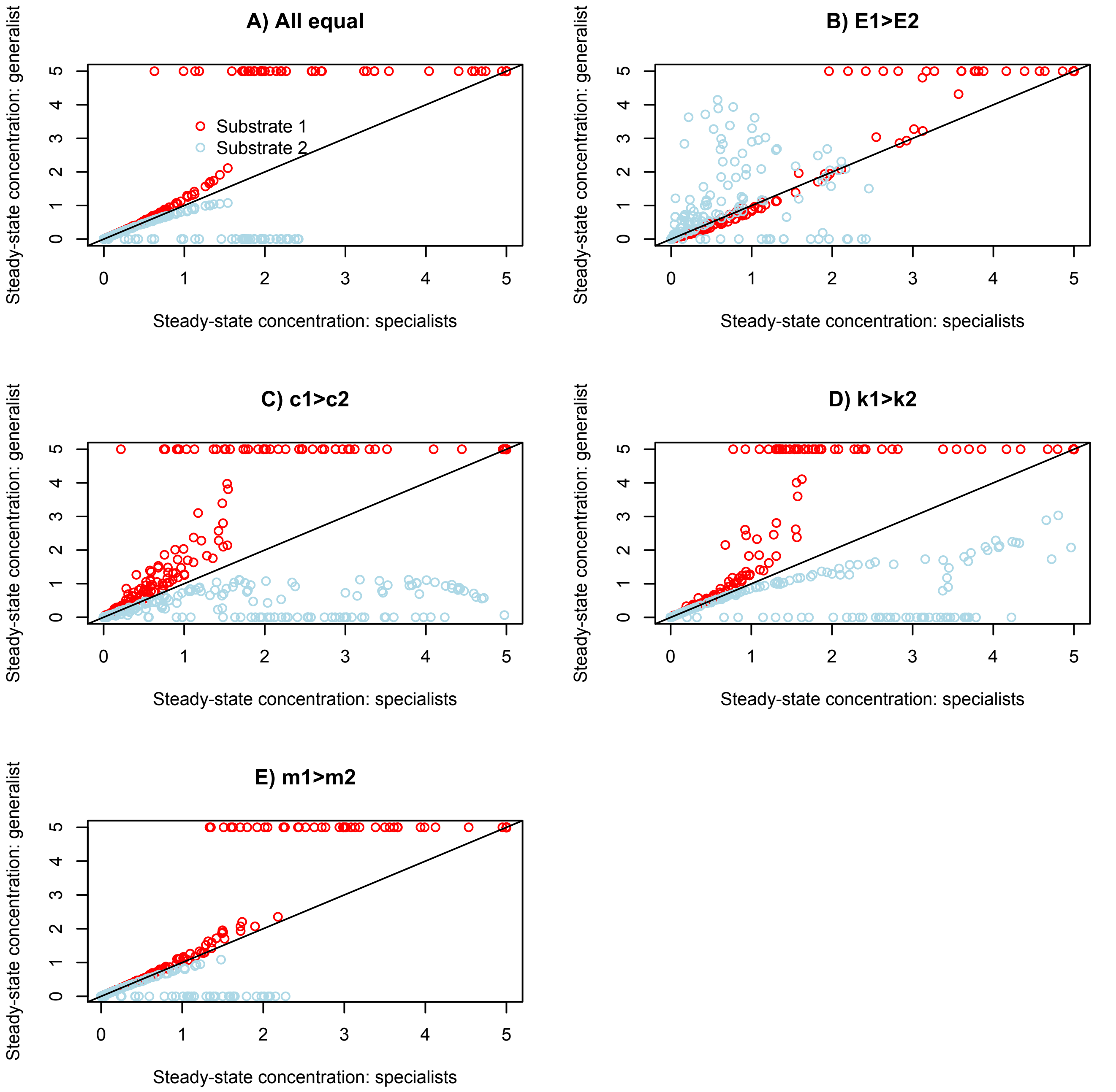
A) All equal
B) E1>E2
C) c1>c2
D) k1>k2
E) m1>m2
Substrate 1
Substrate 2
Steady-state concentration: generalist
Steady-state concentration: specialists
0
1
2
3
4
5
